# Adverse events beyond the RUBY trial: reporting immunotherapy-associated myocarditis, myositis, and myasthenia gravis in a real-world endometrial cancer case

**DOI:** 10.1016/j.gore.2025.101950

**Published:** 2025-09-08

**Authors:** Yasmin Abozenah, Blair McNamara, Michelle Greenman, Emily Daigle, John Paul Mikhaiel, Daniel DiCapua, Jennifer Kwan, Masoud Azodi, Gary Altwerger

**Affiliations:** a Yale University School of Medicine, Department of Obstetrics, Gynecology and Reproductive Science, Division of Gynecologic Oncology, New Haven, CT 06520, United States; b Yale University School of Medicine, Department of Neurology, New Haven, CT 06520, United States; c Yale University School of Medicine, Department of Internal Medicine, Division of Cardiovascular Medicine, Gynecology and Reproductive Science, New Haven, CT 06520, United States

**Keywords:** Endometrial cancer, Immune checkpoint inhibitors, Dostarlimab, Myasthenia gravis, Myocarditis, Myositis, MMM syndrome, Gynecologic oncology

## Abstract

**Objectives:**

To report a rare case of immune checkpoint inhibitor (ICI)-associated myocarditis, myositis, and myasthenia gravis (MMM syndrome) in a patient with advanced endometrial cancer treated with dostarlimab, highlighting the diagnostic and therapeutic challenges, in addition to the importance of biomarker-informed treatment selection.

**Methods:**

We report the case of a 75-year-old woman with unresectable stage IIIC2 endometrial adenocarcinoma and mismatch repair (MMR) deficiency treated with carboplatin and paclitaxel, followed by dostarlimab. After initiation of dostarlimab, the patient presented with neuromuscular and cardiac symptoms, leading to the diagnosis of myocarditis, myositis, myasthenia gravis (MMM) syndrome requiring hospitalization. A multidisciplinary care including neurologic, cardiac, and critical care management was required.

**Results:**

The patient developed MMM syndrome 81 days after starting dostarlimab, presenting with bilateral ptosis, dysphagia, diplopia and respiratory compromise. Evaluation revealed elevated troponins, elevated creatine kinase, and a positive acetylcholine receptor antibody. Cardiac MRI confirmed myocarditis. Management included high-dose steroids, mycophenolate, IVIG, abatacept, and tofacitinib, with initial stabilization.

**Conclusions:**

MMM syndrome is a rare but life-threatening complication of ICI therapy. Early recognition and multidisciplinary management are crucial. This case underscores the importance of weighing the risks and benefits of initiating immunotherapy and utilizing biomarker-driven clinical decisions.

## Highlights

• First reported case of myocarditis, myositis, myasthenia gravis (MMM) syndrome in endometrial cancer following dostarlimab.
• MMM syndrome have been described in melanoma and lung cancer, but not in gynecologic malignancies.
• Early recognition and multidisciplinary management of irAEs are critical for patient outcomes.

## 1 Introduction

Endometrial cancer is the most common gynecologic malignancy in developed countries. For advanced or recurrent disease, standard treatment includes surgery and chemotherapy, often with carboplatin and paclitaxel (Miller et al., 2020). The addition of immune checkpoint inhibitors (ICIs), particularly in mismatch repair-deficient tumors, is now standard of care and a promising strategy.

Dostarlimab, a PD-1 inhibitor, has been shown to improve survival in endometrial cancer, particularly in the dMMR population. In the RUBY trial (Mirza et al., 2023), the hazard ratio for progression-free survival (PFS) in the dMMR subgroup was 0.28 (95 % CI, 0.16–0.50), reflecting a 72 % reduced risk of progression or death. However, ICIs are associated with immune-related adverse events (irAEs) due to enhanced T-cell activation. These irAEs can affect any organ, including the heart, muscles, and neuromuscular junction, manifesting as myocarditis, myositis, and myasthenia gravis (MMM syndrome) (Vijayan et al., 2021).

While MMM syndrome has been documented in melanoma, non-small cell lung cancer, and colorectal cancer, such adverse events remain unreported in gynecologic oncology, to the best of our knowledge (Vijayan et al., 2021, Johnson et al., 2021). Here, we present a unique case of MMM syndrome induced by dostarlimab in a patient with advanced endometrial cancer.

## 2 Case presentation

### 2.1 Patient information

A 75-year-old female with a history of hypertension, hypothyroidism, and morbid obesity was newly diagnosed with advanced-stage FIGO grade 2 endometrioid endometrial adenocarcinoma. She had no family history of cancer and no prior history of autoimmune disorders.

### 2.2 Initial treatment and surgical course

The patient presented with postmenopausal bleeding and was diagnosed with endometrial cancer via hysteroscopy, dilation and curettage. Imaging revealed metastasis to the *para*-aortic lymph nodes. Biopsy of the *para*-aortic lymph nodes confirmed dMMR status (loss of MLH1 and PMS2 by promoter hypermethylation). She was initiated on chemotherapy with carboplatin, paclitaxel, and dostarlimab.

### 2.3 Onset of adverse events and diagnostics

81 days after initiating dostarlimab, the patient presented to the emergency department with acute neurological symptoms, including severe dysphagia, bilateral ptosis, diplopia, voice hoarseness, and generalized muscle weakness. A neurological evaluation revealed fatigable weakness suggestive of myasthenia gravis (MG), further supported by significantly elevated acetylcholine receptor antibodies (AChR Ab: 8.83 nmol/L). MRI of the brain was negative for acute stroke, and electromyography (EMG) was deferred due to the patient's compromised respiratory status. Creatine kinase (CK) was elevated at 412 U/L.

Concurrently, cardiac work up was initiated due to elevated high-sensitivity troponin levels (ranging from 886 to 907 ng/L) and abnormal ECG findings, including intermittent left and right bundle branch blocks. Transthoracic echocardiography (TTE) showed preserved ejection fraction, but cardiac MRI confirmed the diagnosis of myocarditis, consistent with immune checkpoint inhibitor (ICI)-associated cardiac toxicity.

### 2.4 Management

A multidisciplinary team assisted in patient's care within hours of her admission. Neurology and Neurocritical Care specialists managed the immune-related myasthenia gravis and respiratory support in the Neurointensive Care Unit. Cardiology and Cardio-Oncology teams addressed the immune checkpoint inhibitor-associated myocarditis and guided the immunosuppressive treatments. Pulmonary and Critical Care specialists managed respiratory complications due to the neuromuscular weakness, and the Internal Medicine team coordinated daily inpatient care. This collaborative approach was essential in navigating the complex, overlapping adverse effects of MMM syndrome.

On Day 1 of admission, the patient was treated with high-dose intravenous methylprednisolone (1 g/day for 3 days), and adjunctive immunosuppressive therapy was initiated with mycophenolate (1 g twice daily). A single dose of abatacept (10 mg/kg intravenously) was also administered on Day 1 of admission as recommended by the cardiology team for the myocarditis. On Day 2, the patient was started on intravenous immunoglobulin (IVIG) at a dose of 0.4 g/kg/day for five consecutive days to treat the myositis and myasthenia gravis, as recommended by the neurology team. This was followed by switching the IV methylprednisolone to oral prednisone on Day 4, starting at 1 mg/kg/day. On Day 9, the cardio-oncology team increased mycophenolate to 1.5 g twice daily and oral prednisone to 1.5 mg/kg/day in response to rising troponin levels.

Despite this regimen, the patient’s high-sensitivity troponin levels remained persistently elevated for 20 days, with a peak value of 901 ng/L. Due to the persistent increase in troponin levels, on day 20 of admission, the cardiology team started the patient on tofacitinib (5 mg orally twice daily), which led to an immediate decline in troponin levels. Despite initial treatment, the patient’s condition continued to decline, with worsening respiratory compromise requiring transfer to the Neurointensive Care Unit. There, she was started on BIPAP for acute respiratory failure and subsequently stabilized. Steroid therapy was maintained throughout. As her condition improved, she was transferred back to the medical floor and ultimately discharged to a rehabilitation facility with plans for follow-up with gynecologic oncology. At the time of discharge, imaging showed no evidence of disease. Unfortunately, she was later readmitted due to worsening respiratory status and ultimately chose to transition to hospice care.

## 3 Discussion

This case highlights a rare but serious triad of immune-related adverse events, myocarditis, myositis, and myasthenia gravis (MMM syndrome), in a patient receiving dostarlimab for endometrial cancer. While MMM syndrome has been previously been described in other malignancies such as melanoma, non-small cell lung cancer, and colorectal cancer (Vijayan et al., 2021, Johnson et al., 2021, Suzuki et al., 2022), reports are notably absent in current gynecologic oncology literature, including the RUBY trial (Mirza et al., 2023). This case, to our knowledge, is the first to document MMM syndrome in gynecologic oncology.

Early recognition of MMM syndrome remains clinically challenging due to its nonspecific and overlapping symptoms, which may mimic sepsis, stroke, or cancer progression. In this case, elevated troponin levels and confirmatory cardiac MRI established the diagnosis of myocarditis. Concurrent myositis was suggested by significantly elevated creatine kinase levels. Myasthenia gravis was suspected based on clinical findings and supported by markedly elevated acetylcholine receptor antibody levels, although electromyography (EMG) could not be performed due to the patient’s respiratory compromise.

Despite the early initiation of high-dose corticosteroids, the patient's condition was refractory to standard immunosuppressive therapy. This led to an escalation in management to tofacitinib, a JAK inhibitor, which resulted in a biochemical response as seen by declining troponin levels. This case highlights the importance of rapid, multidisciplinary management and early consideration of second-line immunosuppressive agents in steroid-refractory cases. Guidelines by the American Society of Clinical Oncology (ASCO) support the use of therapies such as mycophenolate mofetil and abatacept, managing severe irAEs (Schneider et al., 2018), with JAK inhibitors demonstrating efficacy in steroid-refractory cases of ICI myocarditis (Kwan et al., 2023). Prompt discontinuation of ICI therapy and early initiation of aggressive immunosuppression are essential to mitigate potentially fatal complications of MMM syndrome. However, outcomes remain unpredictable, with a recent systematic review reporting an in-hospital mortality rate of 38 % (Lipe et al., 2024).

Importantly, this case underscores the need for a thoughtful, individualized risk–benefit discussion with the patient and care team prior to initiating immune checkpoint inhibitor (ICI) therapy. This is particularly relevant as ICIs may be used in both MMR-deficient and −proficient patients, who have differing efficacy outcomes but similar potential for immune-related toxicities. In patients with deficient MMR (dMMR) and advanced or residual uterine cancer, as in this case presentation, the benefit of ICIs is well established, supported by trials like RUBY and GY018. However, the efficacy of ICIs in patients with high grade cancers and proficient MMR (pMMR) status remains less robust. These toxicity discussions are held alongside conversations about the potential benefits of immunotherapy, reinforcing a shared decision-making approach that prioritizes patient autonomy. Moreover, the patients and families must demonstrate full awareness and understanding of the potential risks and benefits before treatment initiation.

In the RUBY trial, patients with dMMR endometrial cancer treated with dostarlimab plus chemotherapy had a progression-free survival (PFS) benefit of 67 % compared to chemotherapy-only group (HR 0.33; 95 % CI, 0.18–0.63). In the pMMR subgroup, the PFS benefit was not as pronounced, with a 26 % improvement in PFS (HR 0.74; 95 % CI, 0.57–0.97) (Powell et al., 2024). Similarly, the GY018 trial demonstrated a clinical and statistically significant PFS benefit in the dMMR subgroup of 70 % (HR 0.30; 95 % CI, 0.19–0.48), but when analyzing the pMMR subgroup (HR 0.54; 95 % CI, 0.41–0.71) the benefits were modest at 46 and a 4.4-month improvement in PFS. Although the pMMR subgroup demonstrated a statistically significant improvement in these studies, clinically these therapeutic options are not as beneficial when compared to the dMMR groups (Eskander et al., 2023, Powell et al., 2024). These results reinforce that checkpoint inhibition offers the greatest benefit in dMMR endometrial cancer, where the benefit-to-risk ratio is favorable. In contrast, because pMMR tumors derive less benefit while carrying the same toxicity risk, the risk may outweigh the benefit in this group depending on the patient’s comorbidities and their desires.

These nuances highlight the importance of biomarker-informed decision-making and informed conversations with patients about the potential for both possible benefits and serious, potentially life-threatening immune-related adverse events. The overarching principle must be to ensure that the benefit outweighs the risk, especially when the clinical margin between the two is narrow; predictive biomarkers can help in these situations.

Considering this, careful case-by-case counseling remains essential. At our institution, patients are counseled on both the common side effects of immunotherapy and the rare but potentially severe grade 3–4 immune-related adverse events. Common irAEs include fatigue (20–25 %) and arthralgia/myalgia (10–15 %). Other frequently reported events also include hypothyroidism (10–15 %), hyperthyroidism (5–7 %), rash/pruritus (15–20 %), diarrhea/colitis (8–12 %), hepatitis (5–8 %), and pneumonitis (3–5 %). Severe grade 3–4 irAEs are less common overall, with 7–10 % reported in RUBY trial. Meningitis and encephalitis were reported at < 1 %. Notably, the life-threatening complication of MMM syndrome has no reported percentages in gynecologic cancers.

These findings highlight an important principle in oncology: proposed treatment regimens must demonstrate a clear and meaningful benefit when weighed against their potential risks. Historically, introducing therapies into less-studied populations with limited evidence of efficacy has sometimes led to their later restriction due to clear harm. This serves as a cautionary note to apply new therapies carefully, particularly in the setting of a pMMR biomarker, counseling patients on the modest benefits while acknowledging the potential for immune-related toxicities. In the context of immunotherapy, a biomarker-informed decision-making and careful patient counseling is important, particularly in the setting of pMMR tumors where the benefit is not as robust and the toxicity risk remains.

This case, despite the patient developing MMM syndrome, underscores that the therapeutic benefit outweighed the risks, given the rarity of this complication. The care team reviewed both common and rare ICI toxicities with the patient; however, MMM was not discussed due to the absence of reports in the gynecologic literature. In the context of a dMMR tumor, ICI counseling should emphasize a favorable benefit–risk profile while also addressing the potential for MMM given its life-threatening nature. This stands in contrast to pMMR tumors, where the benefits are more modest and the toxicity risks may approach the therapeutic gain. The strength of this benefit in dMMR disease is illustrated in the PFS reported in the RUBY trial. Importantly, patients and families must be informed of the differential benefits added in a dMMR tumor versus a pMMR tumor and the continued risk of toxicity.

## 4 Conclusion

MMM syndrome is a rare but severe immune-related toxicity associated with ICIs such as dostarlimab. Early recognition is critical, especially as combination chemotherapy–immunotherapy regimens become more prevalent in gynecologic oncology. It is also important to discuss the potential benefits and risks of ICI therapy with the patient and family in the context of MMR predictive biomarker status, given that ICI toxicities can be life-threatening. Moreover, in this patient population, it is important to maintain a high index of suspicion for ICI-adverse events to expediate treatment if needed. Care teams should not hesitate to start these patients on steroids even if diagnostic work up is not complete yet, as early intervention with steroids before a confirmed diagnosis can be lifesaving.

## Consent

The patient has succumbed to her disease before the writing of this case report.

## CRediT authorship contribution statement

**Yasmin Abozenah:** Writing – original draft, Resources. **Blair McNamara:** Writing – review & editing. **Michelle Greenman:** Writing – review & editing. **Emily Daigle:** Writing – review & editing. **John Paul Mikhaiel:** Writing – review & editing. **Daniel DiCapua:** Writing – review & editing, Validation. **Jennifer Kwan:** Writing – review & editing, Validation. **Masoud Azodi:** Writing – review & editing. **Gary Altwerger:** Writing- original draft, Writing – review & editing, Validation, Conceptualization.

## Declaration of competing interest

The authors declare that they have no known competing financial interests or personal relationships that could have appeared to influence the work reported in this paper.
